# Firmicutes-enriched IS*1447* represents a group of IS*3*-family insertion sequences exhibiting unique + 1 transcriptional slippage

**DOI:** 10.1186/s13068-018-1304-8

**Published:** 2018-11-01

**Authors:** Ya-Jun Liu, Kuan Qi, Jie Zhang, Chao Chen, Qiu Cui, Yingang Feng

**Affiliations:** 1 CAS Key Laboratory of Biofuels, Shandong Provincial Key Laboratory of Energy Genetics, Qingdao Institute of Bioenergy and Bioprocess Technology, Chinese Academy of Sciences, Qingdao, China; 2 Dalian National Laboratory for Clean Energy, Dalian, China; 3 University of Chinese Academy of Sciences, Chinese Academy of Sciences, Beijing, China; 4 Present Address: Department of Biosystems Engineering, Auburn University, Auburn, AL 36849 USA

**Keywords:** Transposable element (TE), Frameshift, Lignocellulose, Thermophilic, *Clostridium thermocellum*

## Abstract

**Background:**

Bacterial insertion sequences (ISs) are ubiquitous mobile genetic elements that play important roles in genome plasticity, cell adaptability, and function evolution. ISs of various families and subgroups contain significantly diverse molecular features and functional mechanisms that are not fully understood.

**Results:**

IS*1447* is a member of the widespread IS*3* family and was previously detected to have transposing activity in a typical thermophilic and cellulolytic microorganism *Clostridium thermocellum*. Phylogenetic analysis showed that IS*1447*-like elements are widely distributed in Firmicutes and possess unique features in the IS*3* family. Therefore, IS*1447* may represent a novel subgroup of the IS*3* family. Unlike other well-known IS*3* subgroups performing programmed − 1 translational frameshifting for the expression of the transposase, IS*1447* exhibits transcriptional slippage in both the + 1 and − 1 directions, each with a frequency of ~ 16%, and only + 1 slippage results in full-length and functional transposase. The slippage-prone region of IS*1447* contains a run of nine A nucleotides following a stem-loop structure in mRNA, but mutagenesis analysis indicated that seven of them are sufficient for the observed slippage. Western blot analysis indicated that IS*1447* produces three types of transposases with alternative initiations. Furthermore, the IS*1447*-subgroup elements are abundant in the genomes of several cellulolytic bacteria.

**Conclusion:**

Our result indicated that IS*1447* represents a new Firmicutes-enriched subgroup of the IS*3* family. The characterization of the novel IS*3*-family member will enrich our understanding of the transposition behavior of IS elements and may provide insight into developing IS-based mutagenesis tools for thermophiles.

**Electronic supplementary material:**

The online version of this article (10.1186/s13068-018-1304-8) contains supplementary material, which is available to authorized users.

## Background

Transposable elements (TEs) are ubiquitously present in nature [1]. They can change their position in the genome and play critical roles in genome function and evolution [2, 3]. The bacterial insertion sequences (ISs) are the simplest TEs that have essential impacts on genome evolution and expression [4]. ISs generally have a length of 0.7–2.5 Kb, containing inverted repeat (IR) sequences at both termini and one or two open reading frames (Orfs) to encode cognate transposase (Tpase) [5]. The Tpases can recognize the IRs and then catalyze cleavage at the IS ends, followed by IS transfer into the target site via a cut-and-paste or copy-and-paste mechanism. A short flanking, directly repeated (DR) duplication is usually generated at the insertion site during the DNA strand transfer [5–8]. In addition, some IS elements can implement the recoding of their Orfs via a programmed frameshifting strategy at the transcriptional or translational level [5, 9, 10]. In this way, a single DNA fragment can encode different functional Tpases. By controlling the expression intensity and interactions of the Tpases, the transposition activity and specificity of the IS may be regulated [11].

Various ISs are classified into 29 families in the ISfinder Database (https://www-is.biotoul.fr/index.php) [12] based on their different transposition chemistry, IR and DR sequence features, Orf organizations, and the nature of their target sequences [13]. The composition and order of the functional domains of the encoded Tpases are also used for IS classification. The IS*3* family is one of the largest and best-studied of the IS families and is further divided into 5 main subgroups, including IS*2*, IS*3* [14], IS*51* [15], IS*407* [16], and IS*150* [17], based on the alignment of Orf sequences [13]. The members of the IS*3* family have generally conserved IR, terminating with the dinucleotide 5′-CA-3′, and express Tpases via programmed − 1 ribosomal frameshifting [5]. The copy-and-paste transposition mechanism of the IS*3* family has also been addressed through the extensive analysis of IS*911*, a member of the IS*3* subgroup [8, 11, 18–21].

Most of the known IS elements have been derived from mesophilic bacteria rather than thermophiles, according to ISfinder [12]. Identification of thermophilic IS elements has been achieved largely through genomic annotations, rather than from experimental evidence. Although several IS elements have been discovered in thermophilic *Caldicellulosiruptor* and *Clostridium* species in the active form, their transposition mechanisms have not been further revealed [22–24]. However, thermophiles are of great interest in industry, because of their unique biochemistry and thermostable enzymes. The importance of thermophilic bacteria in biorefineries has recently been proposed [25]. For example, *Clostridium thermocellum* (also named *Ruminiclostridium thermocellum* or *Hungateiclostridium thermocellum*) is considered a promising biocatalyst in industrial biorefineries for lignocellulosic biomass utilization, and targeted genetic engineering has been widely performed on this cellulolytic and anaerobic thermophile [26–31]. Hence, the detection and functional analysis of thermophilic IS elements may promote greater understanding of the physiology of thermophiles and support the development of thermostable genetic tools.

We discovered an active IS element, IS*1447,* that could mutate a thymidine kinase (Tdk) gene by insertion when Tdk was used as the counterselection marker during the genomic editing in the *C. thermocellum* DSM1313 strain [26]. IS*1447* was also detected in a mutated *cipA* gene of another *C. thermocellum* strain ATCC27405 through previous chemical mutagenesis [24]. Genomic analysis revealed fifteen and eighteen copies of the IS*1447* element in *C. thermocellum* DSM1313 and ATCC27405 genomes, respectively. Additionally, sixteen copies of IS*1447* were detected in the genomes of two other *C. thermocellum* strains, DSM2360 and AD2, according to recently improved sequencing results [32]. This implied high transposition activity and potential functional importance. IS*1447* was annotated as an IS*150* subgroup member of the IS*3* family [17], but we found unique sequence features and a + 1 transcriptional slippage pattern in this study. Phylogenetic analysis also showed that IS*1447*-like elements are widespread in the phylum Firmicutes and not closely related to those from non-Firmicutes species.

## Results

### Discovery of the transposable element IS*1447* in *C. thermocellum* DSM1313

We previously developed a seamless genome editing system for *C. thermocellum* DSM1313 using the thymidine kinase gene *tdk* as a counterselection marker [26]. Theoretically, the Tdk cassette-carrying plasmid should be cured by the host cell in the presence of 10 μg/mL 5-fluoro-2-deoxyuradine (FUDR) because Tdk can convert FUDR to toxic fluoro-dUMP and give rise to cell death by blocking pyrimidine biosynthesis [33, 34]. However, during mutant screening, a majority of the colonies grown on plates with FUDR still contained the transformed plasmid, indicating that Tdk was not functional for counterselection [26].

**Fig. 1:**
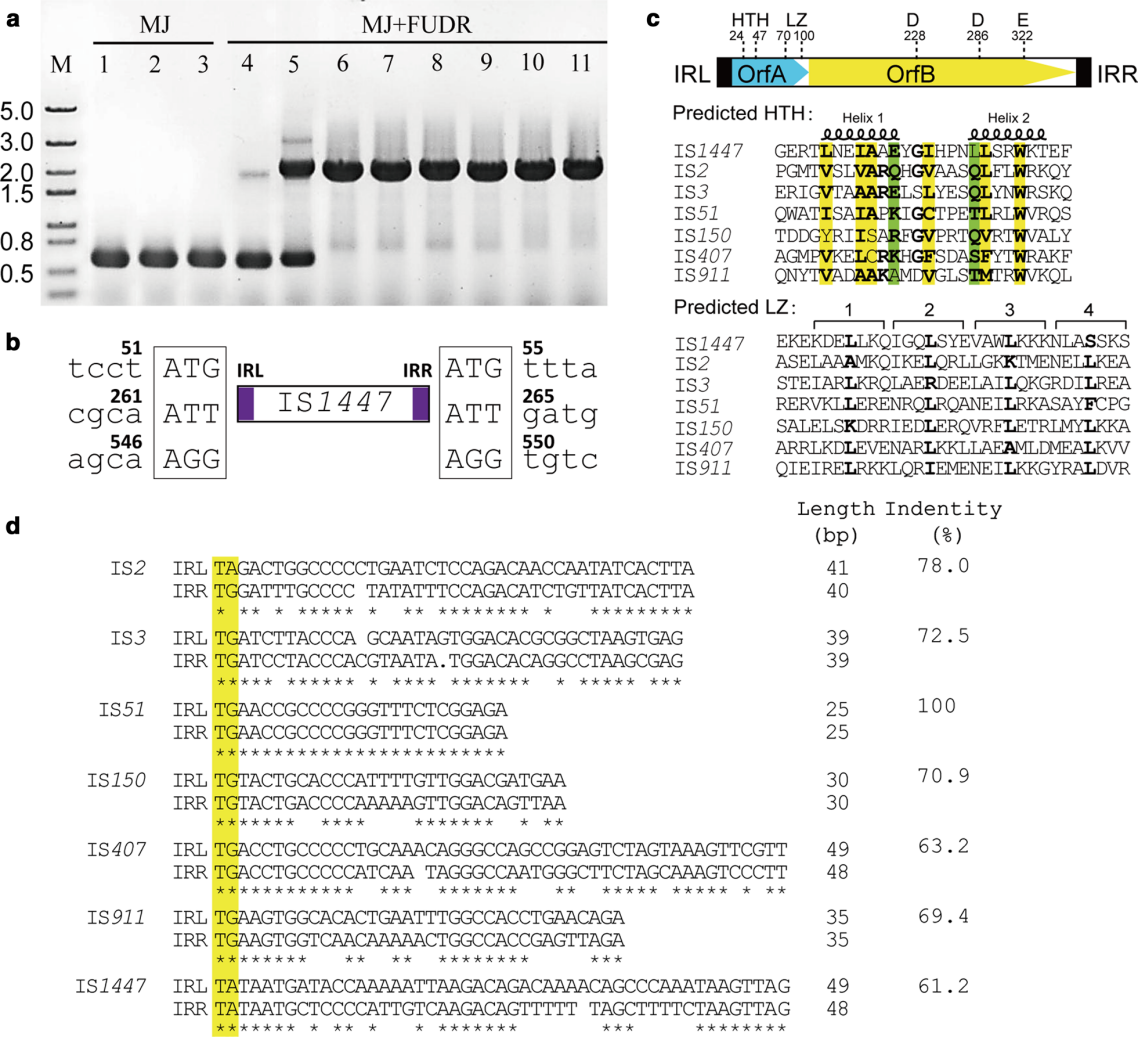
The inserted mutation of the *tdk* gene by IS*1447* and its sequence features. **a** Diagnostic PCR confirmation of the inserted mutation of the *tdk* gene. The transformant Δ*pyrF*::pHK-HR-CglT [26] was continuously cultured in the liquid MJ medium without or with FUDR through 1–3 (lane 1–3) or 1–8 (lane 4–11) subculturings, respectively. The 2-kb band referring to the mutated *tdk* gene became more dominant along with increased subculturings. M, DNA marker. **b** Schematic representation of the IS*1447* insertion in the *tdk* sequence during the counterselection of *C. thermocellum* mutant with the presence of FUDR. Three types of IS*1447* insertion were detected, and the insertion positions were indicated by the coordinates based on the available Tdk-encoding sequence (1–582 bp, Teth514_0091). The duplicated DR sequences were boxed, and the IRs of IS*1447* were in purple. **c** Predicted domain structures of IS*1447*. The putative encoding regions of OrfA and OrfB are indicated by blue and yellow, respectively. The terminal IRs are shown as black boxes. IRL and IRR indicate the left and right IR sequences, respectively. The helix-turn-helix (HTH), leucine zipper (LZ), and DDE motifs are shown with the positions in amino acid residues. Sequence alignments were performed for the HTH and LZ motifs of IS*1447* Tpases with representative members of the major subgroups of the IS*3* family. For the HTH motif, consensus hydrophobic and hydrophilic residues are yellow and green highlighted, respectively. Residues that fit the consensus are in bold. The helix structures are indicated above the sequence. For the LZ motif, the four-component heptad repeats are indicated by the numbers above the sequence, and the leucine repeats are in bold. **d** IRs of IS*1447*, IS*911* and defining members of IS*3*-family subgroups. The dinucleotides at the 5′ terminal are yellow-highlighted. The sequence lengths and the identities between IRL and IRR are indicated to the right of the figure

To confirm the Tdk function during FURD screening, we cultivated the transformants in liquid MJ medium with or without the addition of FUDR continuously through 8 or 3 subculturings, respectively, and then tested the integrity of the *tdk* gene by colony PCR using primers tdk-F/R (Additional file 1: Table S1). After successive cultivation without FUDR, the size of band was consistent with that of the *tdk* gene (580 bp), but both 580-bp and ~ 2-Kb bands were detected when FUDR was present. The 2-Kb band became increasingly dominant along with increased subculturings, and the 580-bp band indicating the wild-type *tdk* gene was not observed after 3 subcultures (Fig. 1a). Sequencing results suggested that the *tdk* gene was mutated by the insertion of IS*1447*, an endogenous TE with a sequence length of 1447 bp, which was also detected in *C. thermocellum* ATCC27405 during previous chemical mutagenesis [24]. Thus, the result confirmed that IS*1447* was an active IS element of the thermophilic microorganism.

### IS*1447* is abundant in the genome of several lignocellulosic species and has unique sequence features

Genome mining showed that *C. thermocellum* DSM1313 has fifteen IS*1447* copies (Clo1313_1104, Clo1313_1298, Clo1313_1865, Clo1313_1651, Clo1313_0773, Clo1313_1935, Clo1313_2369, Clo1313_2700, Clo1313_0507, Clo1313_0508, Clo1313_1640, Clo1313_1641, Clo1313_2656, Clo1313_2663, and Clo1313_2007). Except for Clo1313_2656, which lacks an 8-nt stretch in the middle of the sequence, most of the copies are full-length genes with high sequence identity. Clo1313_2007 and Clo1313_1640, however, are inserted by Clo1313_2008 and Clo1313_1641, thereby encoding IS*256* and another IS*1447* Tpase, respectively (Table 1). The sequences upstream and downstream of the IS*1447* insertions often appear to be pseudogenes or hypothetical genes (Table 1). This indicated that IS*1447* might be involved in the mutation of these once-functional genes. The genomes of *C. thermocellum* strains usually contain multiple IS*1447* copies. For example, eighteen IS*1447* copies were discovered in the genome of *C. thermocellum* ATCC27405 as previously reported [24]. Sixteen IS*1447* copies were found for the genomes of *C. thermocellum* DSM2360 and AD2, the genomes of which have recently been well sequenced and improved [32]. The genotypes of *C. thermocellum* strains can even be differentiated based on the locus and copy numbers of IS*1447* [35]. Furthermore, high copy numbers of IS*1447*-like elements were also detected in the genome of other lignocellulosic species. For example, *C. clariflavum* ATCC19732 and *C. cellulolyticum* H10 are typical thermophilic and mesophilic cellulolytic strains, respectively, while 10 and 11 copies of the IS*1447*-like element encoding full-length Tpases of 400 and 383 amino acids were detected for them, respectively. In addition, 15, 9, and 4 copies of the IS*1447* subgroup ISs were detected during the genomic mining of *Clostridium* sp. Bc-iso-3, *Herbinix hemicellulosilytica* DSM 29228^T^ and *C. stercorarium* subsp. *stercorarium* DSM 8532, respectively, all of which are known cellulose-degrading strains. This result indicated the high abundance and transposition activity of IS*1447*-like elements in several lignocellulosic species [36].

**Table 1:** The IS*1447* genes and relevant up- and downstream genes in the *C. thermocellum* DSM1313 genome

IS*1447* genes	DR	Upstream	Downstream
Clo1313_1104	AAT	Permease	Hypothetical
Clo1313_1298	CAT	Fibronectin	Fibronectin
Clo1313_1865	GAT	Hypothetical	IS*256*/pseudo
Clo1313_1651	AAC	DNA binding domain	Hypothetical/crisper region
Clo1313_0773	AAT	Hypothetical	Pseudo/hypothetical
Clo1313_1935	CTG	Pseudo/hypothetical	Pseudo/hypothetical
Clo1313_2369	None	Pseudo/hypothetical	Pseudo/hypothetical
Clo1313_2700	ATT	Pseudo/hypothetical	Pseudo/hypothetical
Clo1313_0507	AAAA	Pseudo/hypothetical	IS*3*
Clo1313_0508	GTT	Pseudo/hypothetical	IS*3*
Clo1313_1640*	AAT	Crisper region	Pseudo
Clo1313_1641	AAAT	Pseudo	Crisper region
Clo1313_2656	CTG	Pseudo/hypothetical	Pseudo/hypothetical/IS*116*
Clo1313_2663	CCT	Pseudo/hypothetical	IS*4*/hypothetical
Clo1313_2007*	None	Hypothetical	ADP-ribosylation/crystallin J1

* Clo1313_2007 is inserted by Clo1313_2008 encoding an IS*256* transposase in the same direction; Clo1313_1640 is inserted by another IS*1447* transposase-encoding gene Clo1313_1641 in the reverse direction

Nucleotide sequence analysis showed that IS*1447* is a member of the IS*3* family under the name IS*120* (https://www-is.biotoul.fr/index.php) [12]. Like other members of the IS*3* family, the IS*1447* element is primarily occupied by two consecutive open reading frames (ORF), to code for potential Tpases, which contain the essential structures of a helix-turn-helix (HTH) motif, a leucine zipper (LZ) motif and a DDE domain (Fig. 1c). Additionally, the IS*1447* copies in the genome are usually flanked by 3–4 bp directly repeated duplications (DR), except for Clo1313_2369 and Clo1313_2007, which have no DR sequences (Table 1), and the transposition of IS*1447* to the *tdk* gene also generated 3-bp DR sequences of the target DNA, according to the sequencing results (Fig. 1b). However, IS*1447* also presented diverse features from known IS*3*-family members. According to previous phylogenetic analyses, the predicted OrfA and OrfB proteins of IS*1447* are not closely related to any major subgroups of the IS*3* family [13]. The IRs of IS*1447* have a 5′-TA-3′ dinucleotide at the 5′ terminal instead of the conserved dinucleotide 5′-TG-3′ (Fig. 1d). The imperfect IS*1447* IRs of different lengths [49 and 48 bp for the left (IRL) and right (IRR) IR sequence, respectively] share 61.2% identity, which is lower than that of known IS*3* family IRs (Fig. 1d). An insertion sequence IS*Ppy1* has similar sequence features with IS*1447* in terms of IRs [37], but its evolutionary relationship is not close to IS*1447* (see below). This indicated that IS*1447* may represent a novel IS*3*-family subgroup that has a diverse phylogenetic relation with known subgroups.

### IS*1447* represents a novel subgroup that is Firmicutes-enriched

**Fig. 2:**
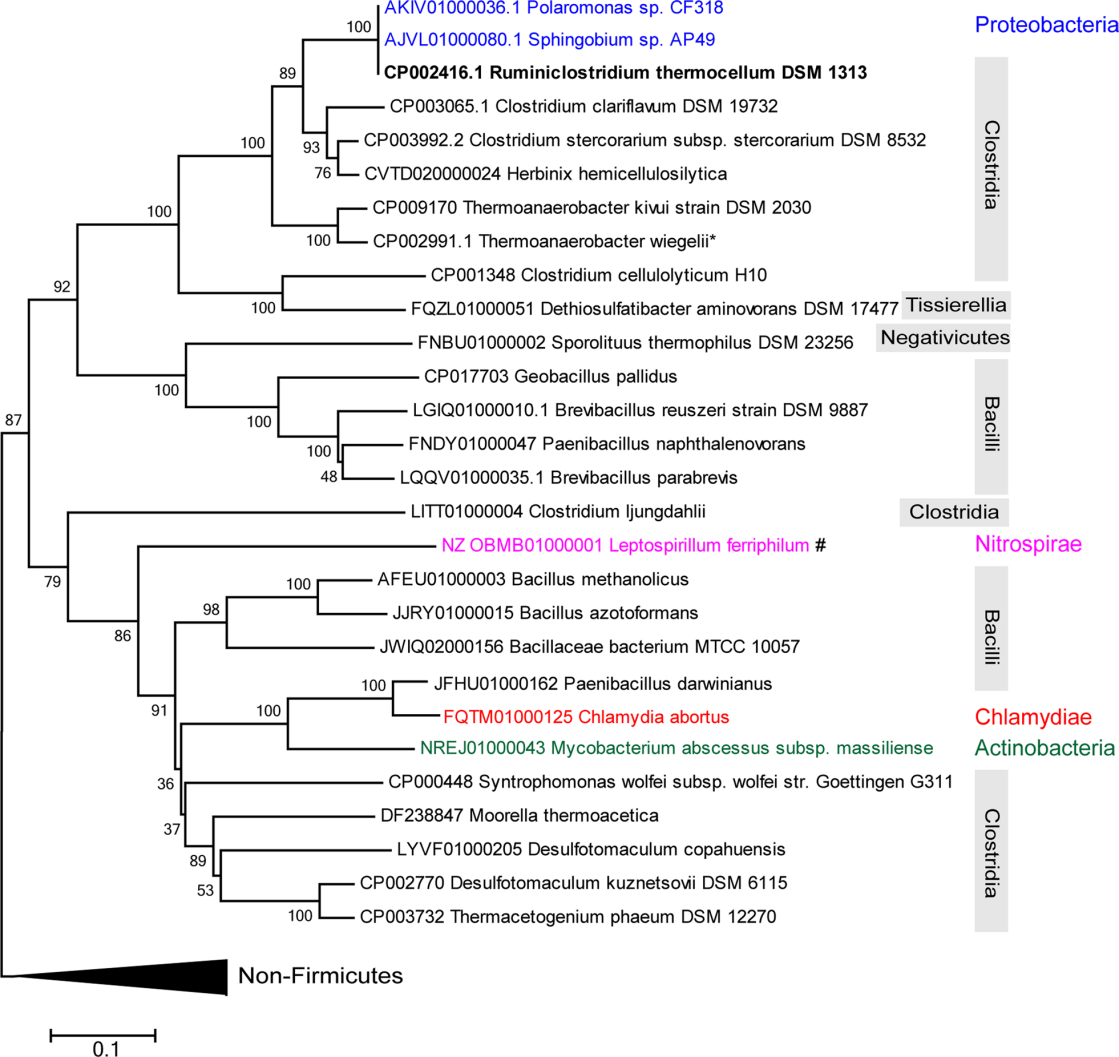
Evolutionary relationships of representative IS*1447*_like OrfAB protein sequences. The evolutionary history was inferred using the Neighbor-Joining method [60]. The optimal tree with the sum of branch length = 13.53466248 is shown. The percentage of replicate trees in which the associated taxa clustered together in the bootstrap test (1000 replicates) are shown next to the branches [38]. The tree is drawn to scale, with branch lengths in the same units as those of the evolutionary distances used to infer the phylogenetic tree. The bar indicates 0.1 estimated changes per amino acid. Most of the sequences from non-Firmicutes species are clustered into one branch as shown in Additional file 1: Figure S1, which is compressed as a black triangle in this figure. The sequence from *C. thermocellum* DSM1313 (also named as *Ruminiclostridium thermocellum* DSM1313 or *Hungateiclostridium thermocellum* DSM1313) is in bold. Non-Firmicutes sequences that are clustered into the Firmicutes branch and their phylum-level affinitions are shown with color. The class-level affinitions of the sequences are shown in gray boxes to the right of the Figure. The accession numbers of the nucleotide sequences are given in front of the species names. The strains with OrfAB proteins that are translated via no or − 1 slippage are marked by the asterisk or pound sign, respectively. The nucleotide and amino acid sequences of the IS*1447*-like genes that perform + 1 transcriptional slippage are listed in Additional file 2: Table S3

To investigate the distribution of IS*1447*-like elements, BLASTp alignment was performed using the amino acid sequence of IS*1447*-encoded OrfAB (GenBank Accession Number ADU74917) as the reference. 2111 significant hits were detected with a sequence coverage and identity of over 50% and 35%, respectively, from 695 organisms (693 from 18 bacterial phyla and 2 archaeal species). Sixty-two sequences were randomly selected to represent different phyla for phylogenetic analysis. The selected OrfAB-like proteins from Firmicutes (28 sequences) and non-Firmicutes (34 sequences) strains were generally separated into two branches of the phylogenetic tree (Fig. 2 and Additional file 1: Figure S1).

IS*1447*_OrfAB-like Tpases were widespread in the two main Classes, Clostridia, and Bacilli, of the phylum Firmicutes and were also detected in the Classes Negativicutes and Tissierellia (Fig. 2). High bootstrap values in the Firmicutes branch indicated closed relationships with IS*1447* from *C. thermocellum* of the IS*1447*_OrfAB-like Tpases (Fig. 2). Compared to the Firmicutes branch, the non-Firmicutes branch showed low bootstrap values, indicating low confidence and high variability of the evolutionary relationships [38] (Additional file 1: Figure S1). In addition, nucleotide sequence analysis showed that a large proportion (76.5%, 26 of 34 randomly selected sequences) of the insertion sequences from the non-Firmicutes branch exhibited no or − 1 frameshifting for the expression of Tpases, including the IS*Ppy1* element from the plasmid pKLH80 of *Psychrobacter maritimus* MR29-12 (GenBank Accession Number AM992204), which was determined previously to display a subgroup of the IS*3*-family [37] (Additional file 1: Figure S1). Thus, IS*1447*-like elements are primarily present in the phylum Firmicutes and not closely related to those from non-Firmicutes species. IS*1447* can be considered to represent the Firmicutes-enriched insertion sequences belonging to a new subgroup of the IS*3* family.

Interestingly, five IS*1447*_OrfAB-like Tpases from *Proteobacteria*, *Nitrospirae*, *Chlamydiae*, and *Actinobacteria* were detected in the Firmicutes branch (Fig. 2), in which the ones from the *Alphaproteobacteria* strain *Sphingobium* sp. AP49 and *Betaproteobacteria* strain *Polaromonas* sp. CF318 had 100% sequence identity with the IS*1447*_OrfAB from *C. thermocellum* DSM1313, while IS*1447*_OrfAB-like proteins from *Chlamydiae* and *Actinobacteria* strains had a close relationship with those from *Bacilli* strains. This indicated that, in addition to vertical gene evolution, horizontal gene transfer of the IS*1447*-like TEs may also occur among bacterial species.

### IS*1447* exhibits + 1 transcriptional slippage instead of − 1 ribosomal frameshifting

**Fig. 3:**
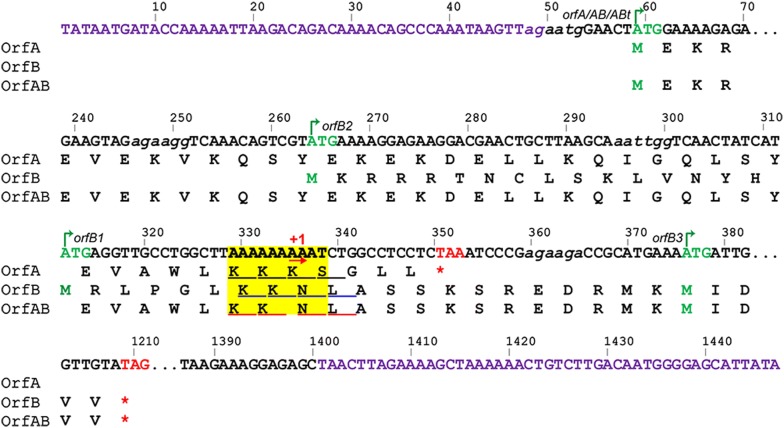
The nucleotide sequence of IS*1447.* The nucleotides are numbered above the sequence. The putative IR sequences are in purple. The putative initiation and termination codons of the transposase(s) are in green and red, respectively. The italic letters in lower case indicate potential ribosome-binding sites. The green arrows above the sequence indicate the nucleotides from which the cloning sequences for the expression of OrfA, OrfAB, OrfABt, and OrfB proteins (OrfB1, OrfB2, and OrfB3) were selected. The potential slippage-prone region is highlighted in yellow, and the reading frames for OrfA, OrfB, and OrfAB are underlined in black, blue, and red, respectively

The well-characterized subgroups of the IS*3* family generally produce three types of Tpases by − 1 frameshifting within a A_6_G/C/A motif at the translational level [13]. However, unlike other subgroups, the expression of OrfAB of IS*1447* requires + 1 frameshift (Fig. 3) [13]. Nucleotide sequence analysis indicated that IS*1447* has a run of nine A nucleotides at the 3′ end of the OrfA reading frame, which may be a slippage-prone region for rearrangement at the transcriptional level [39–41].

To investigate whether IS*1447* exhibits transcriptional slippage in *C. thermocellum*, the transcript sequences of the potential slippage-prone region were determined by cloning. The total mRNA of *C. thermocellum* DSM1313 was isolated and reverse transcribed to obtain cDNA, which was further used as the template to amplify the IS*1447* sequence containing the potential slippage-prone region. The genomic DNA was also isolated and used as the template for PCR using the same primers simultaneously. The PCR products derived from the cDNA and the control DNA were cloned and sequenced. For the cDNA, 8 and 9 of 52 randomly selected clones had ten and eight A nucleotides in the potential slippage-prone region, respectively, and the other clones showed nine A nucleotides (Additional file 1: Figure S2). In contrast, all 30 clones derived from the control genomic DNA showed nine A nucleotides. The result showed that the IS*1447* element exhibits transcriptional rearrangement in both the − 1 and + 1 directions, while only the + 1 transcriptional slippage may result in the expression of a full-length Tpase. The dissociation and reassociation of the nascent RNA with its DNA template within a transcribing RNA polymerase complex may cause transcriptional rearrangement, and the slippage occurred efficiently at the location of homopolymeric runs of A or T nucleotides [40, 42]. The dual-direction slippage of IS*1447* may be explained by the instability of the A–U rich RNA–DNA hybrid.

### IS*1447* requires a run of seven A nucleotides for transcriptional slippage

**Fig. 4:**
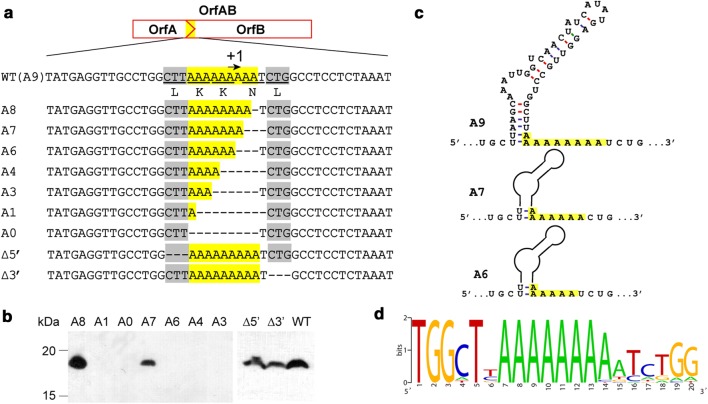
Construction and analyses of the IS*1447* elements with a mutated slippage-prone region. **a** Potential frameshift window sequences of IS*1447* mutants. The A_9_T-related sequences are highlighted in yellow. The putative reading frames and + 1 frameshift sites for OrfAB are underlined and indicated above the sequence, respectively. Three-nucleotide reading frame sequences were deleted from the wild-type IS*1447* (A_9_T) to construct mutants A_7_ (A_9_T lacking AAT), A_6_ (A_9_T lacking AAA), A_4_ (A_9_T lacking A_5_T), A_3_ (A_9_T lacking A_6_), A1 (A_9_T lacking A_8_T), and A0 (A_9_T lacking A_9_); ∆5′ and ∆3′ indicate the mutants with deleted 5′ and 3′ codons to the A_9_T motif, which are gray highlighted. **b** Western blot analysis of IS*1447* mutants using Anti-His6-tag antibody. M, protein standards, the molecular weights are shown to the left of the figure. **c** The predicted secondary structures of IS*1447* mRNA sequences around the slippage-prone region. A stem-loop structure is predicted to include two A nucleotides of the A_9_U sequence (yellow highlighted). The RNA secondary structures were predicted by mfold web server (http://unafold.rna.albany.edu/?q=mfold/RNA-Folding-Form) [55]. **d** The consensus sequences of the slippage-prone region of IS*1447*-like TEs

As shown above, IS*1447* produced the fused OrfAB protein via + 1 transcriptional slippage within a run of nine A nucleotides (Fig. 3 and Additional file 1: Figure S2). To verify the key region for + 1 slippage, various OrfABt mutants were constructed by deleting 3-nucleotide reading frames from the A_9_T sequence (Fig. 4a and Additional file 1: Table S2). Immunoblotting analyses showed that both the wild-type OrfABt with the A_9_T sequence and the positive control OrfABt-A_8_ produced a His-tag-bearing protein of approximately 18.8 kDa, indicating the OrfABt protein produced via + 1 slippage (Fig. 4b). RNA structure prediction indicated that the mRNA of IS*1447* contains a stem-loop structure adjacent to the slippage-prone region (Fig. 4c). The mutant carrying a string of seven successive A nucleotides showed the same OrfABt band, indicating that the lack of an AAT sequence did not influence slippage. Other mutants with zero to six A nucleotides showed no band referring to frameshift proteins (Fig. 4b). These results suggested that the + 1 slippage of IS*1447* required a string containing at least seven repeated A nucleotides. The slippage-prone sequences of 34 IS*1447*-like genes (Additional file 2: Table S3) that perform + 1 transcriptional slippage were aligned and analyzed by WebLogo [43]. The result indicated that the + 1 slippage-prone region had a conserved run of seven to nine A nucleotides (Fig. 4d). Hence, although it has been suggested that the minimum length of the A or T run to promote transcriptional rearrangement is nine [39], IS*1447* required only seven A nucleotides for + 1 transcriptional slippage. Additionally, a modified stem-loop structure could be formed without the 3′ or 5′ codon to the A_9_T sequence, and the slippage was not apparently influenced (Fig. 4b, c).

### IS*1447* produces three types of Tpases with alternative initiations

**Fig. 5:**
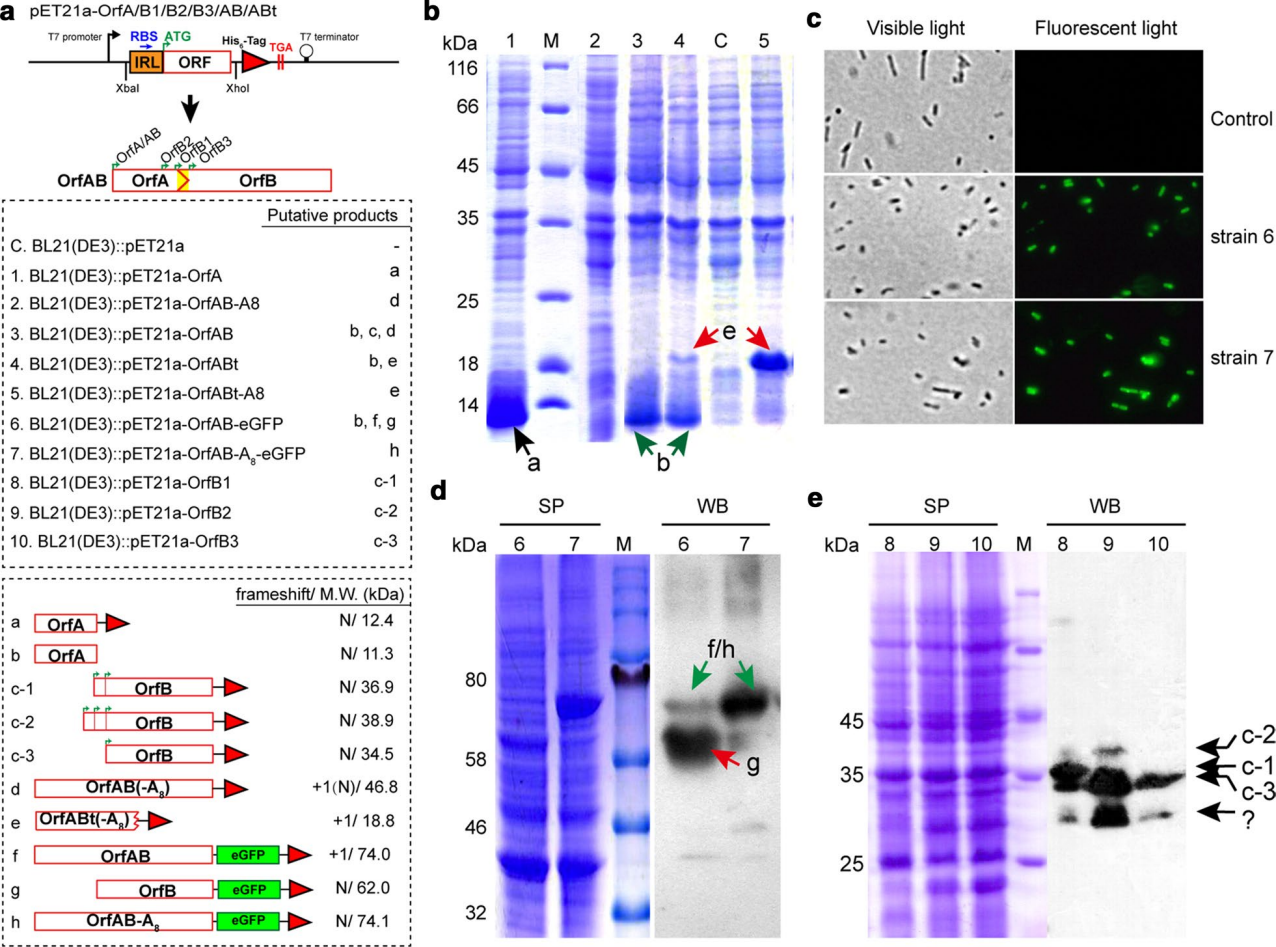
The expression of Tpases by IS*1447*. **a** Schematic representation of the pET21a-derived plasmids constructed for Tpase expression. Green arrows and red double lines indicate the potential initiation and termination codons for Tpase translation, respectively. Red triangles indicate the His_6_-tag. The orange box indicates the IRL sequence of IS*1447*, and the putative RBS in IRL is indicated by a blue arrow. The frameshift region is highlighted in yellow. The constructed *E. coli* BL21(DE3) strains, their predicted products with (+ 1) or without (N) programmed slippage and theoretical molecular weights are listed in the dashed boxes below. Green boxes indicate the eGFP gene. All lanes and bands indicated by arrows in **b**, **c**, and **d** are named according to the strains and products shown in the dashed boxes. **b** SDS-PAGE analysis of the crude extracts of the *E. coli* strains expressing IS*1447* Tpases. BL21(DE3)::pET21a was used as the negative control. Black, green, and red arrows indicate the bands corresponding to the products a, d, and e, respectively, as shown in **a**. The ~ 18.8 kDa protein produced by BL21(DE3)::pET21a-OrfABt-A_8_ was further confirmed by mass spectrometry (Additional file 1: Figure S3). **c** Fluorescent imaging of strains expressing the eGFP-bearing IS*1447* Tpases. Control, strain 6 and strain 7 refer to those listed in **a**. **d** SDS-PAGE (SP) and immunoblotting (WB) analyses of the expression of IS*1447* Tpases-eGFP fusion proteins. Green and red arrows indicate the bands of approximate 74 and 62 kDa corresponding to the products g and f/h, respectively, as shown in **a**. **e** SDS-PAGE (SP) and immunoblotting (WB) analyses of the expressed OrfB proteins (OrfB1, OrfB2, and OrfB3) in *E. coli* BL21(DE3). Black arrows indicate the bands referring to the putative OrfB proteins expressed with different initiation codons as shown in **a**. M, protein standards

T7 RNA polymerase-driven expression in *E. coli* BL21(DE3) was further performed to investigate the production of the IS*1447* Tpases via the + 1 slippage. The potential ribosome-binding site (RBS) involved in the IRL sequence was used to mimic the protein translation pattern in *C. thermocellum* (Fig. 3). All produced proteins contained six successive histidines at the C-terminus for affinity purification or immunoblotting (Fig. 5a). Previous studies indicated that IS*911*, a model for mechanistic analysis of the IS*3* family, produced both full-length and truncated OrfAB proteins, and the one lacking the catalytic domain became more abundant at elevated temperature [44]. Hence, a 151-amino acid OrfAB derivate protein truncated for the DDE domain was constructed as well and termed OrfABt. The artificial Tpases OrfAB-A_8_ and OrfABt-A_8_ were produced as positive controls for further analyses by deleting a nucleotide A from the A_9_T string. As shown in Fig. 5b BL21(DE3)::pET21-OrfABt produced a protein of the same size with OrfABt-A_8_ (approximately 18.8 kDa). The protein was further verified to be the truncated OrfABt protein by mass spectrometry (Additional file 1: Figure S3). BL21(DE3)::pET21-OrfABt also produced a smaller protein of approximately 11 kDa, which may refer to the OrfA protein. This result indicated that the truncated OrfAB could be expressed via + 1 slippage in *E. coli*. However, for the OrfAB-expressing strains BL21(DE3)::pET21-OrfAB and BL21(DE3)::pET21-OrfAB-A_8_, only the bands referring to OrfA were observed, and no apparent expression of full-length OrfAB was detected.

The gene encoding an enhanced green fluorescent protein (*eGFP*) was ligated at the 3′ terminal of the *orfAB* sequence to test the expression of the full-length Tpase. The fused protein OrfAB-A_8_-eGFP was also expressed as the positive control. Bright green fluorescence was observed for the *E. coli* strains expressing either OrfAB-eGFP or OrfAB-A_8_-eGFP (Fig. 5c). Because OrfA/OrfAB and OrfB are in the relative translational reading phases 0 and + 1, respectively (Fig. 3), the fused expression of eGFP with OrfB required no slippage. Considering the possibility that the fluorescence is caused by the OrfB-eGFP protein (Fig. 5a), the expressed proteins were also analyzed by Western blotting using the Anti His_6_-Tag antibody (Fig. 5d). Compared to the positive control OrfAB-A_8_-eGFP, a 74-kDa band was clearly detected for BL21(DE3)::pET21-OrfAB-eGFP by immunoblotting but in lower abundance, indicating the successful expression of the fused protein OrfAB-eGFP. Additionally, an ~ 62-kDa band was detected in high abundance, which is the size expected for a fusion protein OrfB-eGFP (Fig. 5a). These results indicated that the IS*1447* produced three types of Tpase, OrfA, OrfB, and the full-length OrfAB, simultaneously.

To define the open reading frames and translation initiation of the IS*1447* Tpases, Ni^2+^-affinity chromatography was employed to purify OrfA, OrfB, and OrfABt proteins for N-terminal sequencing. BL21(DE3)::pET21a-OrfA and BL21(DE3)::pET21a-OrfABt were used to express the OrfA and OrfABt proteins, respectively, in which OrfABt was analyzed instead of OrfAB because of the low expression level of OrfAB. N-terminal sequencing showed that OrfA and OrfABt shared the same initiation sequence of “MEKRK,” which was consistent with bioinformatics analysis (Fig. 3). Nucleotide sequence analysis showed that OrfB might have three putative initiation codons with potential RBS sequences (Fig. 3). Thus, three *E. coli* strains, BL21(DE3)::pET21a-OrfB1, -OrfB2, and -OrfB3, were constructed accordingly to express OrfB proteins OrfB1, OrfB2, and OrfB3 with different theoretical molecular weights (M.W.), respectively (Figs. 3 and 5a). However, the OrfB purifications failed because of extremely low (undetectable) expression, and OrfB N-terminal sequencing was not possible. Immunoblotting analysis, however, confirmed that BL21(DE3)::pET21a-OrfB1, -OrfB2, and -OrfB3 produced proteins of 36.9, 38.9, and 34.5 kDa, respectively (Fig. 5e), which fit their theoretical molecular weights. Interestingly, all OrfB-expressing strains produced the OrfB3 protein of 34.5 kDa (Fig. 5e). Thus, the ATG at 375 bp of the IS*1447* may be the main initiation codon of OrfB (Fig. 3). In this case, the reading frames of OrfA and OrfB have no overlapping region, which is different from known IS*3* members that perform frameshifting [13, 19]. Additionally, a band of approximately 30 kDa was also detected in all OrfB-expressing strains, indicating an alternative initiation codon of OrfB (Fig. 5e). However, no ATG codon was observed downstream of the 375-bp ATG in the IS*1447* sequence, and the expression of the 30-kDa protein may be initiated with a non-ATG codon.

## Discussion

The IS elements are the simplest and most numerous transposable elements that can cause bacterial genome rearrangements and evolution [4, 45]. Certain IS elements have been used to construct transposons for genome characterization, mutagenesis, and editing based on extensive understanding of the sequence features and transposition chemistry [46–49]. However, the IS elements from thermophilic microorganisms are currently underexplored. Several thermophilic IS elements have been reported to have transposition activity, such as IS*Cbe4*, IS*Cahy1* and IS*1447* from *Caldicellulosiruptor hydrothermalis*, *Caldicellulosiruptor bescii*, and *Clostridium thermocellum*, respectively [22–24], which provide insight into developing genetic tools for thermophiles based on endogenous genetic elements, such as the development of the Thermotargetron system for gene targeting based on a thermophilic group II intron [50].

The genomes of the *C. thermocellum* strains DSM1313, ATCC27405, DSM2360, and AD2 have high IS*1447* copy numbers, indicating the high transposition activity of the IS element. No IS*1447* sequence was detected for another three *C. thermocellum* strains, YS, BC1, and JW20, likely due to the low quality of the genomic sequences at the contig assembly level, since identification of TEs using the current genome sequencing methods is still a challenge [51]. Multiple copies of IS*1447*–like elements were also detected in several other lignocellulosic species, especially those from phylum Firmicutes. Most of the IS*1447*-like elements contain relatively conserved long IR sequences that are specific compared to other well-known IS*3*-family members. IS*1447* may not generate double-stranded DNA circles as the well-characterized IS*3*-family member IS*911* [11] because no IRL-IRR junction was detected. Most importantly, IS*1447* follows a diverse frameshift pattern of + 1 transcriptional slippage compared to other well-known IS3-family members, which read though full-length Orfs using ribosomal frameshifting at the translation level [41, 52]. Evolutionary relationship analysis has shown that IS*1447* represents a novel IS*3*-family subgroup that is Firmicutes-enriched.

Transcriptional rearrangement was first discovered in *E. coli* to produce β-galactosidase by − 1 transcriptional frameshift resulting from the insertion of an extra A [9]. The transcriptional slippage phenomenon has been widely observed since then and has been predicted for IS elements according to genome annotation [40, 41]. The slippage mechanism has been discussed in previous studies [39, 42, 53, 54]. Two main slippage-prone sequence patterns, X_m_Y_n_ and A(T)_n_, may be involved in bacterial transcriptional realignment. An IS*630* family element from a *Roseiflexus* strain was proved to exhibit transcriptional realignment in the heteropolymeric sequence T_5_C_5_, which fits the X_m_Y_n_ pattern [42]. We proved herein that IS*1447* employs an A(T)_n_ pattern sequence as the slippage-prone region for transcriptional rearrangement. RNA-structure-mediated transcriptional slippage has been proposed for the *Roseiflexus*-IS*630*. The hairpin sequence upstream of the T_5_C_5_ slippage-prone region of IS*630* is important for slippage [42]. As predicted by the mfold web server [55], the mRNA of IS*1447* also contains a stem-loop structure adjacent to the A_9_U region (Fig. 4c), which may play a key role in melting the upstream part of the RNA–DNA hybrid and promoting slippage [42]. Interestingly, two A nucleotides of the A_9_U sequence are involved in the stem-loop structure, which may result in the remaining seven A nucleotide sequences acting as the “true” slippage region. We have proved that the deletion of AAT of the A_9_T sequence showed no significant influence but that further replacement of the seventh A with T completely disrupted the slippage event (Fig. 4b), indicating that the slippage would occur with a run of no less than seven successive A nucleotides. According to previous studies, a run of nine A or T nucleotides is required as the minimum length to promote transcriptional rearrangement [39], and the RNA polymerase requires over seven A or T nucleotides for slippage [41, 53]. For other IS*1447*-like elements that perform + 1 transcriptional slippage, similar stem-loop structures can also be detected, even though they contain diverse 5′-codons adjacent to the slippage region compared to IS*1447* (Additional file 2: Table S3). For IS*1447*, a modified stem-loop structure could be formed without the 3-bp upstream codon to the A_9_T sequence (Fig. 4c). Thus, although the upstream RNA secondary structure may be essential for the slippage of IS*1447*-like elements, the 5′-codon adjacent to the slippage region is not indispensable.

The frameshift frequency of the IS elements could be modulated by the physiological state of the host cells [8]. Hence, the microorganisms may control the expression and combination of different functional Tpase domains [11], as well as the transposition activity of the IS elements [56], by programmed frameshifting at either the transcriptional or translational level. For IS*1447*, transposition activity was only observed in *C. thermocellum* with the presence of exotic stress. For example, we detected the transposition of IS*1447* only in the presence of counterselection stress reagent FUDR, which would cause cell death in this study. Zverlov et al. observed IS*1447* transposition in the genome of *C. thermocellum* ATCC27405 under chemical mutagenetic stress induced by ethyl-methanesulfonate [24]. Wilson et al. also detected the insertion of an IS*3* element, which may be IS*1447*, in the genome of *C. thermocellum* DSM1313 during targeted gene deletions using the hypoxanthine phosphoribosyl transferase gene as the counterselection marker [57]. There could be transposition precedence for the active IS*1447* with the presence of exotic stress. Thus, this could be an efficient method to detect the in vivo transposition and analyze the mechanism of IS*1447* and other transposable elements with the presence of exotic stresses.

*Clostridium thermocellum* has promising industrial potential as a whole-cell catalyst to convert lignocellulose to fermentable sugars, biofuels, and biochemicals. Nevertheless, targeted engineering is still required to enhance its degrading activity and the yield of target products [26, 27, 31]. Because the transposition of IS*1447* may make genetic manipulation difficult, it is necessary to inactivate IS*1447* in *C. thermocellum*. However, it would be difficult or tedious work to delete fifteen highly identical copies of IS*1447* genes in *C. thermocellum* DSM1313. However, IS*1447* elements may be silenced or blocked if its inducing activation mechanism could be identified. Thus, future investigations of the transposition and activation mechanism of IS*1447* will provide valuable information that will enhance our understanding of these intriguing and potentially useful systems.

## Conclusions

Insertion sequences are of great interest in developing transposon-based tools for genome characterization, mutagenesis, and editing. Hence, the sequence features and transposing mechanisms should be extensively understood. Thermophilic IS elements are rarely reported compared to mesophilic ones. Here, we discovered and analyzed an active IS element IS*1447* from a thermophilic bacterium *C. thermocellum,* representing a novel Firmicutes-enriched subgroup of the IS*3* family. Interestingly, the well-known IS*3*-family members usually employ − 1 ribosomal frameshifting for the transposase expression, but IS*1447* exhibits + 1 transcriptional slippage within a region of seven successive A nucleotides, although it is generally considered that RNA polymerase needs more than seven A or T nucleotides for slippage. IS*1447*-subgroup elements are abundant in the genomes of several lignocellulosic bacteria. Thus, the investigation of IS*1447* will enrich our understanding of the transposition behavior of IS elements and may promote the development of IS-based mutagenesis tools for thermophiles.

## Methods

### Bacterial strains and cultivation

Bacterial strains used in this study are listed in Additional file 1: Table S2. *Escherichia coli* strains were cultivated aerobically at 37 °C in Luria–Bertani (LB) liquid medium with shaking at 200 rpm or on solid LB plate with 1.5% agar. *C. thermocellum* strains were grown anaerobically at 55 °C in MJ medium [58] with 5 g/L cellobiose as the carbon source. 30 μg/mL chloramphenicol and 100 μg/mL ampicillin were supplemented to the medium when necessary.

### Phylogenetic analysis

Phylogenetic analyses were performed with the MEGA5 software (version 5.05, [59]). The nucleotide sequences encoding the IS*1447*-like OrfAB proteins (Additional file 2: Table S4) were retrieved from NCBI, translated in silico, and pre-aligned with the ClustalW algorithm. For proteins that require rearrangement for full-length expression, artificial fusion was performed by manually deleting one nucleotide A from the A_9_T frameshift window without changing the OrfAB amino acid sequence. The full-length OrfAB-like proteins were then aligned with the ClustalW algorithm, and the alignment was refined manually. The DNA sequences were aligned according to the aligned proteins. Phylogenetic trees were calculated based on amino acid sequences of full-length OrfAB-like proteins using the neighbor-joining algorithm [60]. Tree topologies were verified by bootstrap analysis with 1000 replicates. The aligned DNA sequences were analyzed by WebLogo [43] to show the consensus sequences. The affinitions of the organisms containing the IS*1447*_OrfAB-like proteins were identified using the Taxonomy Browser (https://www.ncbi.nlm.nih.gov/taxonomy).

### Nucleic acid isolation and reverse transcription PCR

*Clostridium thermocellum* DSM1313 was grown at 55 °C to mid-log phase with 5 g/L cellobiose as the carbon source. Genomic DNA and total RNA were isolated using Blood & Cell Culture DNA Mini Kit and RNeasy Mini kit (Qiagen), respectively. Reverse transcription was performed using the isolated total RNA as the template with SuperScript III First-Strand Synthesis Supermix (Invitrogen) and random hexamer primers. Both genomic DNA and cDNA were used as templates for PCR with *pfu* DNA polymerase and primer set OrfAB-1/-3 (Additional file 1: Table S1). The isolated RNA was also used for PCR to test the potential contamination of DNA.

### Cloning and sequencing

The obtained PCR products were purified using a Gel extraction Kit (Omega), ligated to a pMD19-T vector (Takara), and transformed into *E. coli* DH5α competent cells according to the manufacturer’s protocol. Thirty and fifty-two colonies were randomly selected from the pools derived from genomic DNA or cDNA, respectively, for sequencing using a universal M13F primer.

### Plasmid construction

All plasmids were constructed based on pET21a (Additional file 1: Table S2) to add a 3′ terminal successive six histidine tag to the target proteins for further purification and immunoblotting. All segments of IS*1447* were amplified from the genome DNA of *C. thermocellum* DSM1313 and verified by sequencing. Restriction sites XbaI and XhoI were used for DNA cloning to eliminate the ribosome-binding site (RBS) of pET21a. Instead, the potential endogenous RBSs of IS*1447* was employed for protein translation. Site-directed mutagenesis was accomplished using the reverse PCRs according to a previous report [61]. To construct plasmids pET21a-OrfAB-eGFP and pET21a-OrfAB-A_8_-eGFP for the fused expression of eGFP with OrfAB and OrfAB-A_8_, respectively, primer set 21-r1/2 was used to linearize pET21a-OrfAB or pET21a-OrfAB-A_8_ through PCR first. The eGFP gene was then amplified by eGFP-o1/2. The primers 21-r1 and 21-r2 contained overlapping regions of eGFP-o1 and eGFP-o2, respectively, and seamless assembly cloning was further performed to ligate the linear plasmids and eGFP genes according to the manufacturer’s protocol (Clone Smarter Technologies).

### Protein expression

The pET21a-derived plasmids were constructed in *E. coli* DH5α, and then transformed into *E. coli* BL21(DE3) for protein expression. The cells were cultivated to the mid-exponential phase (OD_600_ nm = 0.8–1.0), and 1 mM of isopropyl β-d-thiogalactoside (IPTG) was added to initiate the protein expression. The cells were further cultivated at 30 °C for 3 h and were used for fluorescence imaging with a fluorescent microscope BX51 (Olympus, Beijing, China) or for sodium dodecyl sulfate–polyacrylamide gel electrophoresis (SDS-PAGE) analysis.

### Protein analyses

SDS-PAGE was performed to check the protein purity and composition as previously described [62]. The molecular weight of the protein was estimated according to the relative mobility of protein ladders (11–116 kDa or 10–230 kDa, New England BioLabs). The Bradford method was used for protein quantification [63]. The mass spectroscopy analyses were achieved using Maldi-TOF-TOF (Sangon Biotech). For immunoblotting, the SDS–polyacrylamide gel was wet blotted onto a presoaked polyvinylidene difluoride membrane at 400 mA for 1 h in an ice bath. The membrane was then blocked by incubating overnight in TBST buffer (20 mM Tris–HCl, 138 mM NaCl, 0.08% Tween 20, pH 7.6) containing 50 g/L skim milk. Afterward, the membrane was incubated for 2 h at room temperature with anti-His6-tag rabbit IgG according to the manufacturer’s protocol (Sangon Biotech). Then, the membrane was washed three times with TBST buffer, incubated for a further 2 h with a solution of anti-rabbit IgG(Fc) goat IgG conjugated with alkaline phosphatase (Sangon Biotech), washed as before, and visualized using HyGlo HRP ECL Detection Kit (MDBio) in accordance with manufacturers’ protocol.

## Additional files

**Additional file 1: Table S1.** Primers used in this study. **Table S2.** Bacterial strains and plasmids used in this study. **Figure S1.** Evolutionary relationships of representative IS*1447*_like OrfAB protein sequences from non-Firmicutes strains. The evolutionary history was inferred using the Neighbor-Joining method [60]. The optimal tree with the sum of branch length = 13.53466248 is shown. The percentage of replicate trees in which the associated taxa clustered together in the bootstrap test (1000 replicates) is shown next to the branches [38]. The tree is drawn to scale, with branch lengths in the same units as those of the evolutionary distances used to infer the phylogenetic tree. The bar indicates 0.1 estimated changes per amino acid. Sequences from Firmicutes species are clustered into one branch as shown in Fig. 2, which is compressed as a black triangle in this figure. The accession numbers of corresponding nucleotide sequences are given in front of the species names. The strains with OrfAB proteins that are translated via no or − 1 frameshifting are marked by an asterisk or pound sign, respectively. **Figure S2.** Sequencing results of the colonies containing partial IS*1447* sequences amplified from cDNA of *C. thermocellum* DSM1313. The potential slippage-prone regions with − 1, + 1 or no transcriptional rearrangement are yellow highlighted. **Figure S3.** Identification of OrfABt protein by mass spectrometry analysis. The protein produced by BL21(DE3)::pET21a-OrfABt-A_8_ with the size of ~18.8 kDa was investigated (Fig. 5b). Peptides detected by mass spectrometry are in red.

**Additional file 2: Table S3.** The sequence information of the IS*1447*-like genes that perform + 1 transcriptional slippage. **Table S4.** The nucleotide sequences encoding the IS*1447*-like OrfAB proteins for phylogenetic analyses.
