# Supplementary material for: Firmicutes-enriched IS1447 represents a group of IS3-family insertion sequences exhibiting unique + 1 transcriptional slippage
Source: Biotechnol Biofuels. 2018 Nov 1;11:300. doi: 10.1186/s13068-018-1304-8 (PMC6211511; doi:10.1186/s13068-018-1304-8)
Supplement: Supplementary file 1 — Additional file 1: Table S1. Primers used in this study. Table S2. Bacterial strains and plasmids used in this study. Figure S1. Evolutionary relationships of representative IS1447_like OrfAB protein sequences from non-Firmicutes strains. The evolutionary history was inferred using the Neighbor-Joining method [60]. The optimal tree with the sum of branch length = 13.53466248 is shown. The percentage of replicate trees in which the associated taxa clustered together in the bootstrap test (1000 replicates) is shown next to the branches [38]. The tree is drawn to scale, with branch lengths in the same units as those of the evolutionary distances used to infer the phylogenetic tree. The bar indicates 0.1 estimated changes per amino acid. Sequences from Firmicutes species are clustered into one branch as shown in Fig. 2, which is compressed as a black triangle in this figure. The accession numbers of corresponding nucleotide sequences are given in front of the species names. The strains with OrfAB proteins that are translated via no or − 1 frameshifting are marked by an asterisk or pound sign, respectively. Figure S2. Sequencing results of the colonies containing partial IS1447 sequences amplified from cDNA of C. thermocellum DSM1313. The potential slippage-prone regions with − 1, + 1 or no transcriptional rearrangement are yellow highlighted. Figure S3. Identification of OrfABt protein by mass spectrometry analysis. The protein produced by BL21(DE3)::pET21a-OrfABt-A8 with the size of ~18.8 kDa was investigated (Fig. 5b). Peptides detected by mass spectrometry are in red. [file 13068_2018_1304_MOESM1_ESM.docx]

# Additional file 1

**Table S1.** Primers used in this study.

| Primer | Sequence^*^ | Notes |
| --- | --- | --- |
| tdk-F | ATGATATACGGATCTTTG | To test the integrity of gene *tdk* |
| tdk-R | TGTACGGGGAACTTCAT |  |
| OrfAB-1 | GGtctagaTATAATGATACCAAAAATTAAG (XbaI) | To amplify full length OrfAB seuqence |
| OrfAB-2 | GGctcgagCTATACAACTGCCAGTAGCTGG (XhoI) |  |
| OrfAB-o1 | TGCCTGGCTTAAAAAAAATCTGGCCTCCTC | To amplify OrfAB-A_8_T sequence with OrfAB-1 by overlap PCR |
| OrfAB-o2 | GAGGAGGCCAGATTTTTTTTAAGCCAGGCA |  |
| OrfAB-3 | GGctcgagAATTTCATCAATGATACGCTT (XhoI) |  |
| OrfA-2 | GGctcgagGAGGAGGCCAGATTTTTTTTTA (XhoI) | To amplify OrfA with OrfAB-1 |
| OrfB-1 | GGctcgagGCAAATTGGTCAACTATCAT (XhoI) | To amplify OrfB proteins with OrfAB-1 |
| OrfB-2 | GGtctagaGCAAGGAAACTGATGAAGTAG (XbaI) |  |
| OrfB-3 | GGtctagaATCCCGAGAAGACCGCATG (XbaI) |  |
| A1-1  A1-2 | TATGAGGTTGCCTGGCTTACTGGCCTCCTCTAAATC  GATTTAGAGGAGGCCAGTAAGCCAGGCAACCTCATA | Site directed mutation of A_9_T to A by reverse PCR |
| A4-1 | TATGAGGTTGCCTGGCTTAAAACTGGCCTCCTCTAAATC | Site directed mutation of A_9_T to A_3_A by reverse PCR |
| A4-2 | GATTTAGAGGAGGCCAGTTTTAAGCCAGGCAACCTCATA |  |
| A7-1 | TATGAGGTTGCCTGGCTTAaaaaaaCTGGCCTCCTCTAAATC | Site directed mutation of A_9_T to A_6_A by reverse PCR |
| A7-2 | GATTTAGAGGAGGCCAGTTTTtttAAGCCAGGCAACCTCATA |  |
| A3-1 | TATGAGGTTGCCTGGCTTAaatCTGGCCTCCTCTAAATC | Site directed mutation of A_9_T to A_3_T by reverse PCR |
| A3-2 | GATTTAGAGGAGGCCAGaTTTAAGCCAGGCAACCTCATA |  |
| A6-1 | TATGAGGTTGCCTGGCTTAaaaaatCTGGCCTCCTCTAAATC | Site directed mutation of A_9_T to A_6_T by reverse PCR |
| A6-2 | GATTTAGAGGAGGCCAGatttTTTAAGCCAGGCAACCTCATA |  |
| A0-1 | TATGAGGTTGCCTGGCTTtCTGGCCTCCTCTAAATC | Site directed mutation of A_9_T to T by reverse PCR |
| A0-2 | GATTTAGAGGAGGCCAGaAAGCCAGGCAACCTCATA |  |
| ∆5’-1 | TATGAGGTTGCCTGGAAAAAAAAATCTGGCCTCCTCTAAATC | Site directed deletion of 5’ codon to A_9_T |
| ∆5’-2 | GATTTAGAGGAGGCCAGATTTTTTTTTCCAGGCAACCTCATA |  |
| ∆3’-1 | TATGAGGTTGCCTGGCTTAAAAAAAAATGCCTCCTCTAAATC | Site directed deletion of 3’ codon to A_9_T |
| ∆3’-2 | GATTTAGAGGAGGCATTTTTTTTTAAGCCAGGCAACCTCATA |  |
| 21-r1 | acaAGCTCGAGCACCACCACCAC | To obtain linear pET21a-OrfAB(-A_8_T) by reverse PCR |
| 21-r2 | Accaccaccacctacaactgccagtagctggt |  |
| eGFP-o1 | Gtggtggtgctcgagcttgtacagctcgtccat | To amplify eGFP gene containing overlapping region of pET21a-OrfAB(-A_8_T) |
| eGFP-o2 | GTTGTAGGTGGTGGTGGTGGTGGTATGGTGAGCAAGGGCGAG |  |

^*^Restriction sites are shown in lower case and indicated in parentheses.

**Table S2**. Bacterial strains and plasmids used in this study

| Strains/plasmids | Relevant characteristic | Sources/notes |
| --- | --- | --- |
| **Strains** |  |  |
| *E. coli* |  |  |
| DH5α | *f80dlacZΔM15, Δ(lacZYA-argF)U169, deoR, recA1, endA1, hsdR17(rk−, mk+), phoA, supE44, l−, thi-1, gyrA96, relA1* | Transgen Biotech |
| BL21(DE3) | *ompT gal dcm lon hsdSB(rB− mB−) l (DE3 [lacI lacUV5-T7 gene 1 ind1 sam7 nin5])* | Transgen Biotech |
| BL21(DE3)::pET21a | Derived from BL21(DE3), carrying plasmid pET21a as a negative control | This work |
| BL21(DE3)::pET21a-OrfA | Derived from BL21(DE3), carrying plasmid pET21a-OrfA to express OrfA | This work |
| BL21(DE3)::pET21a-OrfB1 | Derived from BL21(DE3), carrying plasmid pET21a-OrfB1 to express OrfB1 | This work |
| BL21(DE3)::pET21a-OrfB2 | Derived from BL21(DE3), carrying plasmid pET21a-OrfB2 to express OrfB2 | This work |
| BL21(DE3)::pET21a-OrfB3 | Derived from BL21(DE3), carrying plasmid pET21a-OrfB3 to express OrfB3 | This work |
| BL21(DE3)::pET21a-OrfAB | Derived from BL21(DE3), carrying plasmid pET21a-OrfAB to express OrfAB via slippage | This work |
| BL21(DE3)::pET21a-OrfAB-A_8_ | Derived from BL21(DE3), carrying plasmid pET21a-OrfAB-A_8_ to express mutated OrfAB with a A_8_ motif as a positive control | This work |
| BL21(DE3)::pET21a-OrfABt | Derived from BL21(DE3), carrying plasmid pET21a-OrfABt to express truncated OrfAB via slippage | This work |
| BL21(DE3)::pET21a-OrfABt-A_8_ | Derived from BL21(DE3), carrying plasmid pET21a-OrfABt-A_8_ to express truncated OrfAB with a A_8_ motif as a positive control | This work |
| BL21(DE3)::pET21a-OrfABt-A_7_ | Derived from BL21(DE3), carrying plasmid pET21a-OrfABt-A_7_ to express mutated OrfABt | This work |
| BL21(DE3)::pET21a-OrfABt-A_6_ | Derived from BL21(DE3), carrying plasmid pET21a-OrfABt-A_6_ to express mutated OrfABt | This work |
| BL21(DE3)::pET21a-OrfABt-A_4_ | Derived from BL21(DE3), carrying plasmid pET21a-OrfABt-A_4_ to express mutated OrfABt | This work |
| BL21(DE3)::pET21a-OrfABt-A_3_ | Derived from BL21(DE3), carrying plasmid pET21a-OrfABt-A_3_ to express mutated OrfABt | This work |
| BL21(DE3)::pET21a-OrfABt-A_1_ | Derived from BL21(DE3), carrying plasmid pET21a-OrfABt-A_1_ to express mutated OrfABt | This work |
| BL21(DE3)::pET21a-OrfABt-A_0_ | Derived from BL21(DE3), carrying plasmid pET21a-OrfABt-A_0_ to express mutated OrfABt | This work |
| BL21(DE3)::pET21a-∆5’ | Derived from BL21(DE3), carrying plasmid pET21a-∆5’ to express mutated OrfABt | This work |
| BL21(DE3)::pET21a-∆3’ | Derived from BL21(DE3), carrying plasmid pET21a-∆3’ to express mutated OrfABt | This work |
| BL21(DE3)::pET21a-OrfAB-eGFP | Derived from BL21(DE3), carrying plasmid pET21a- OrfAB-eGFP to express the fused protein OrfAB-eGFP via slippage | This work |
| BL21(DE3)::pET21a-OrfAB-A_8_-eGFP | Derived from BL21(DE3), carrying plasmid pET21a- OrfAB-A_8_-eGFP to express the fused protein OrfAB-A_8_-eGFP | This work |
| **Plasmids** |  |  |
| pET21a | Expression vector with C-terminal hexahistidine affinity tag | Invitrogen |
| pET21a-OrfA | pET21a derivative for expression of OrfA | This work |
| pET21a-OrfB1 | pET21a derivative for expression of OrfB1 | This work |
| pET21a-OrfB2 | pET21a derivative for expression of OrfB2 | This work |
| pET21a-OrfB3 | pET21a derivative for expression of OrfB3 | This work |
| pET21a-OrfAB | pET21a derivative carrying IS*1447* sequence for expression of OrfAB via slippage | This work |
| pET21a-OrfAB-A_8_ | Derived from pET21a-OrfAB, deleted a nucleotide A from A_9_T motif of IS*1447* | This work |
| pET21a-OrfABt | pET21a derivative carrying truncated IS*1447* sequence for expression of truncated OrfAB via slippage | This work |
| pET21a-OrfABt-A_8_ | Derived from pET21a-OrfABt, deleted a nucleotide A from A_9_T motif of IS*1447* | This work |
| pET21a-OrfABt-A_7_ | Derived from pET21a-OrfABt, deleted AAT from A_9_T motif of IS*1447* | This work |
| pET21a-OrfABt-A_6_ | Derived from pET21a-OrfABt, deleted three A nucleotides from A_9_T motif of IS*1447* | This work |
| pET21a-OrfABt-A_4_ | Derived from pET21a-OrfABt, deleted A_5_T from A_9_T motif of IS*1447* | This work |
| pET21a-OrfABt-A_3_ | Derived from pET21a-OrfABt, deleted A_6_ from A_9_T motif of IS*1447* | This work |
| pET21a-OrfABt-A_1_ | Derived from pET21a-OrfABt, deleted A_8_T from A_9_T motif of IS*1447* | This work |
| pET21a-OrfABt-A_0_ | Derived from pET21a-OrfABt, deleted nine A nucleotides from A_9_T motif of IS*1447* | This work |
| pET21a-∆5’ | Derived from pET21a-OrfABt, deleted upstream CCT of A_9_T motif | This work |
| pET21a-∆3’ | Derived from pET21a-OrfABt, deleted downstream CTG of A_9_T motif | This work |
| pET21a-OrfAB-eGFP | pET21a-OrfAB derivative for expression of the fused protein OrfAB-eGFP | This work |
| pET21a-OrfAB-A_8_-eGFP | pET21a-OrfAB derivative for expression of the fused protein OrfAB-A_8_-eGFP | This work |


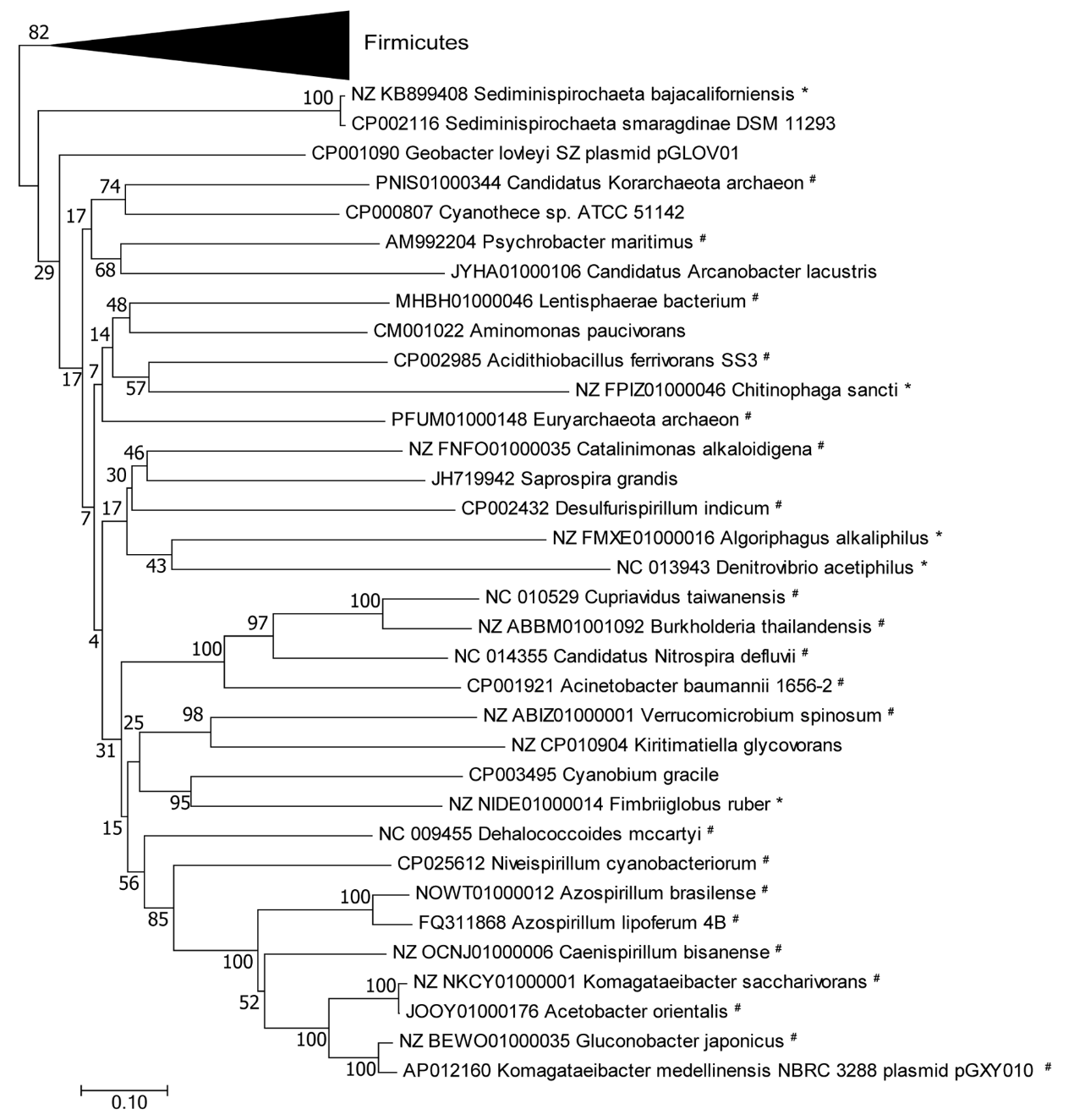


**Figure S1**. Evolutionary relationships of representative IS*1447*_like OrfAB protein sequences from non-Firmicutes strains. The evolutionary history was inferred using the Neighbor-Joining method (1). The optimal tree with the sum of branch length = 13.53466248 is shown. The percentage of replicate trees in which the associated taxa clustered together in the bootstrap test (1000 replicates) are shown next to the branches (2). The tree is drawn to scale, with branch lengths in the same units as those of the evolutionary distances used to infer the phylogenetic tree. The bar indicates 0.1 estimated changes per amino acid. Sequences from Firmicutes species are clustered into one branch as shown in Figure 2, which is compressed as a black triangle in this figure. The accession numbers of corresponding nucleotide sequences are given in front of the species names. The strains whose OrfAB proteins are translated via no or -1 frameshifting are marked by an asterisk or pound sign, respectively.


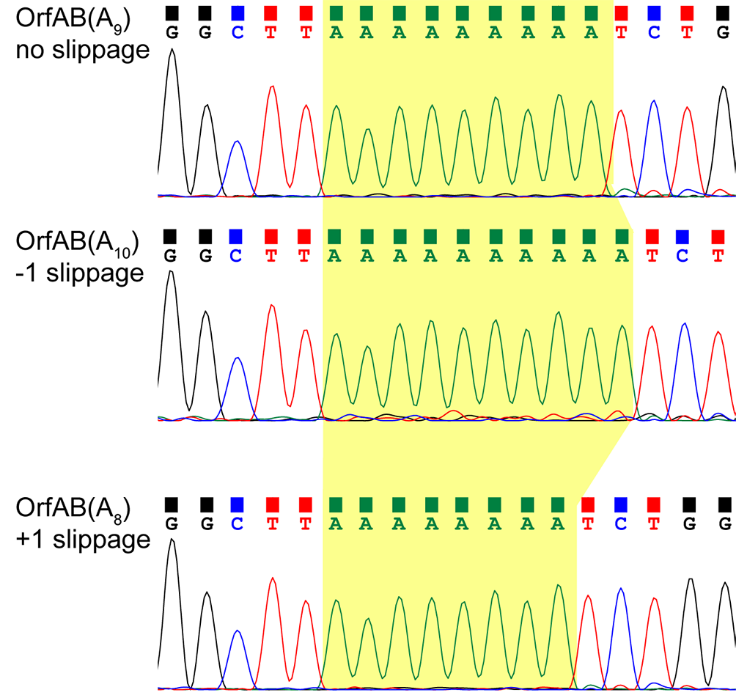


**Figure S2**. Sequencing results of the colonies containing partial IS*1447* sequences amplified from cDNA of *C. thermocellum* DSM1313. The potential slippage-prone regions with -1, +1 or no transcriptional rearrangement are yellow highlighted.


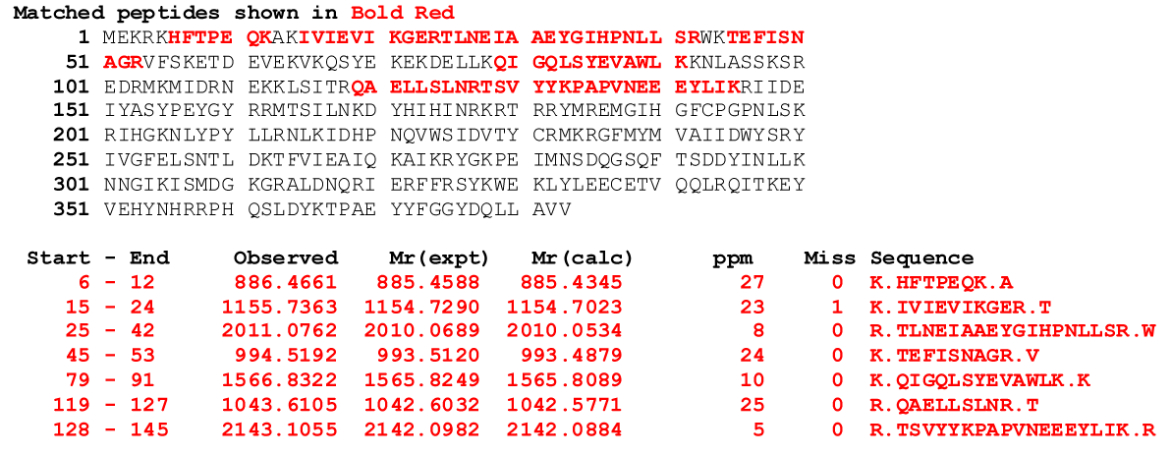


**Figure S3**. Identification of OrfABt protein by mass spectroscopy analysis. The protein produced by BL21(DE3)::pET21a-OrfABt-A_8_ with the size of ~18.8 kDa was investigated (Figure 5b). Peptides detected by mass spectroscopy are in red.

References

1. Saitou, N., and Nei, M. (1987) The neighbor-joining method: a new method for reconstructing phylogenetic trees. *Mol. Biol. Evol.* **4**, 406-425

2. Felsenstein, J. (1985) Confidence limits on phylogenies: An approach using the bootstrap. *Evolution* **39**, 783-791
